# Production and Characterization of Open Cell Cordierite from Boron and Waste Materials by Geopolymer Method for the Emission After Treatment System of Diesel Engines

**DOI:** 10.1002/gch2.202500048

**Published:** 2025-05-13

**Authors:** Yavuz Ergun, Hakan Caliskan, Halil Ibrahim Karali

**Affiliations:** ^1^ Department of Chemical Engineering Faculty of Engineering and Natural Sciences Usak University Usak 64200 Türkiye; ^2^ Department of Mechanical Engineering Faculty of Engineering and Natural Sciences Usak University Usak 64200 Türkiye; ^3^ Department of Motor Vehicles and Transportation Technologies Vocational High School of Technical Sciences Usak University Usak 64200 Türkiye

**Keywords:** after treatment, cordierite, exhaust filter, geopolymer filter, waste recovery

## Abstract

Porous geopolymer materials can be used in various fields such as thermal insulation, filtration, catalyst, and building materials. In this study, open porous geopolymer‐based cordierite materials are produced due to the porous structure, temperature resistance, and easy and low‐cost applications of geopolymer structures, which are oxide ceramic materials that can act as natural catalysts for emission treatment of diesel engines. For the composition of cordierite, waste boron clay, metakaolin, fly ash, and magnesium carbonate are used, while keeping the geopolymerization temperature constant, sodium silicate, sodium hydroxide, polypropylene, and glass fiber, hydrogen peroxide are used to create an alkaline environment. These materials are tested in a 4‐stroke 4‐cylinder diesel engine's exhaust system at 50, 75, and 100 Nm engine torques and 1500, 1700, and 1900 rpm engine speeds. The use of open‐cell geopolymer materials reduces CO emissions by 66%, NOx emissions by 25% and HC emissions by 68%. The open‐cell geopolymer materials are found to be effective in treating over 95% of particulate matter. The chemical and microstructures of the obtained open‐cell geopolymer structures are investigated. It is concluded that the developed products are useful tools for the emission treatments of diesel engines considering the oxidizing and filtering effects of the materials.

## Graphical abstract

Open porous geopolymer‐based cordierite materials are produced due to the porous structure, temperature resistance, easy and low cost applications of geopolymer. For the composition of cordierite, waste boron clay, metakaolin, fly ash and magnesium carbonate are used, while keeping the geopolymerization temperature constant, sodium silicate, sodium hydroxide, polypropylene and glass fiber, hydrogen peroxide are used to create an alkaline environment. These materials are tested in a diesel engine to show the effective emission treatment abilities of the developed products.

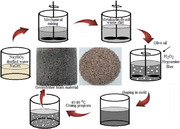

## 1 Introduction

Energy demand increases every year and it shows the interaction between nature and society.^[^ ^1^ ^]^ This increase is expected to cause an increase in the consumption of fossil fuels. Hence, the negative environmental impacts of fossil fuels rise up.^[^ ^2^ ^]^ Emission problems of fossil fuels are important and critical issues for the reputation of internal combustion engines, which threatens their ability to advance significantly and their contribution to reducing emissions.^[^ ^3^ ^]^ To overcome the emission problems of fossil fuel utilization in internal combustion engines, e.g. diesel engines, new structures/materials are produced for the emission treatment.^[^ ^4^, ^5^ ^]^ Especially, porous oxide ceramic structures are being made for many purposes. By using different processes and chemical compositions, porosity, strength, absorbent, filter, and catalyst properties can be added to the material. Especially the chemical composition and pore skeleton (pore shape, geometry, pore size, quantity and size distribution, inter‐pore connectivity, and active surface properties) of porous structures gain importance. Improving porosity in ceramics increases their functionality.^[^ ^6^ ^]^ This functionality is also used in gas‐solid separation and liquid‐solid separation in environmental and other industries due to their high thermal stability, thermal conductivity, and corrosion resistance performance.^[^ ^7^, ^8^ ^]^ Diesel Particulate Filter (DPF), usually made of silicon carbide (SiC)‐based,^[^ ^6^ ^]^ can impart infrared radiation emission properties.^[^ ^10^ ^]^ Regardless of the direction of use of porous cordierite ceramics, especially the low linear expansion coefficient is the most important requirement for the material in the use of catalyst carriers and filtering element technology.^[^ ^11^ ^]^

**Figure 1:**
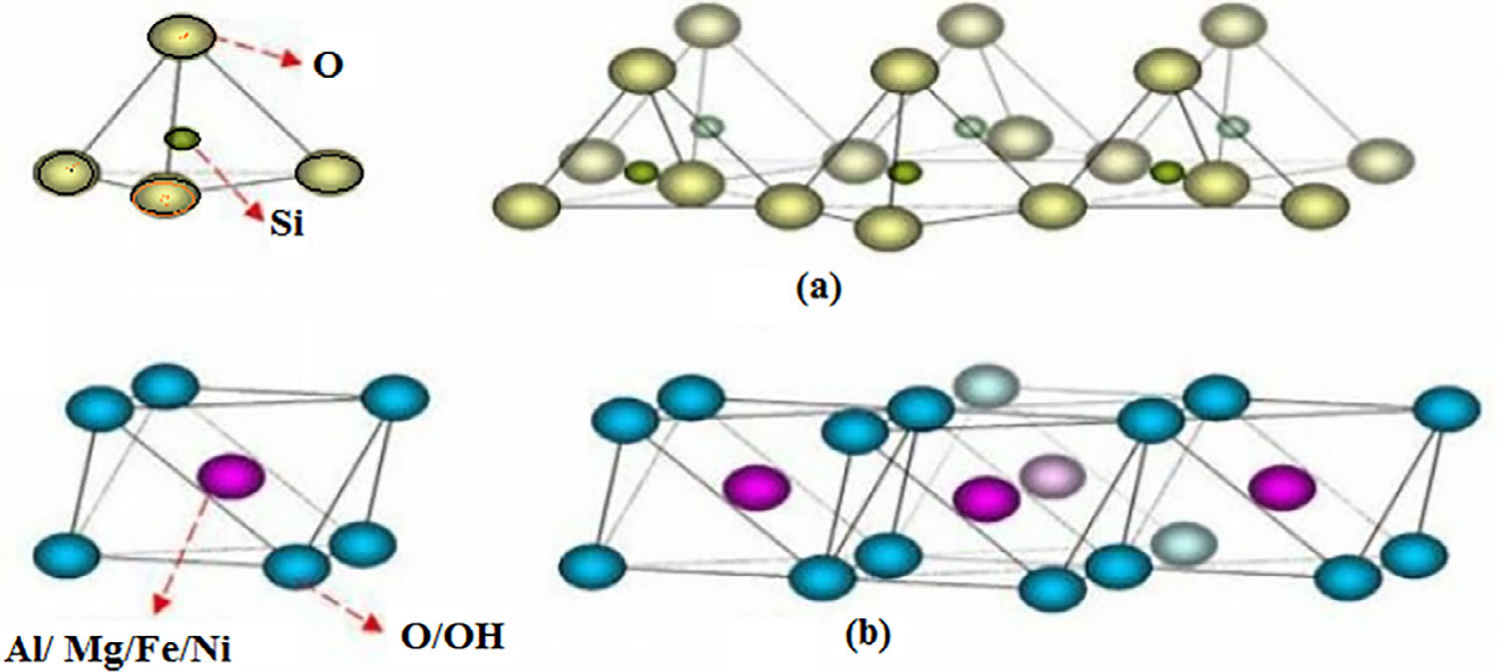
a) Tetrahedron b) Octahedron structure.

Cordierite is an aluminum silicate mineral and crystallizes in three different polymorphs in tetrahedron and octahedron structures in **Figure** **1**. These α‐ β‐ and γ‐ are found in hexagonal, rhombohedral, and monoclinic crystal systems respectively.^[^ ^12^, ^13^ ^]^ Since octahedron structures are more volumetrically larger than tetrahedrons, the Mg in the center has the ability to bond/replace other metals. This changes the physical and surface properties of the material as it allows the load imbalance of the material and the possibility of adding reinforcing elements. Since natural cordierite deposits are scarce, it is produced artificially in the form of sol–gel, co‐precipitation, and high‐temperature solid phase method.^[^ ^14^, ^15^ ^]^ Cordierite is Mg_2_Al_4_Si_5_O_18_ with theoretical chemical weight percentages of 13.7% MgO, 34.9% Al_2_O_3,_ and 51.4% SiO2.

Since cordierite has a wide range of usage areas and can be obtained with alternative production and raw material sources, many studies have been carried out in this field for different purposes in recent years. Among these, we can list porous geopolymer‐based materials.

Geopolymerization involves a process that starts with the reaction of alkaline silicate and alkali salts with alumino‐silicate earth materials at low temperatures.^[^ ^16^ ^]^ In this process, the exothermic chemical process involving dissolution, transport, orientation, and polycondensation (multiple condensation) of molecules in a highly alkaline environment is called geopolymerization.^[^ ^17^ ^]^ Geopolymers are highly porous, low‐density materials with abundant raw material resources, low CO_2_ emission, low energy consumption, low production price, high early strength, and fast strength gain. Thanks to these favorable properties, geopolymers are suitable for engineering use in the construction, automotive, aerospace, metallurgy, and plastics industries. They are also very good in waste management and have even become suitable for use in many similar fields such as art and decoration. One of the areas where geopolymers are widely used is in the treatment of toxic waste and this is becoming increasingly common.

In their study involving the production of geopolymer‐based tube membranes, they reported the successful preparation of porous gradient geopolymer‐based tube membranes with high PM (particulate matter) retention ability.^[^ ^18^ ^]^ PM removal rate for air pollution. Cordierite was synthesized by replica method using polyurethane foams by activation of metakaolin, MgO, and fly ash raw materials in an alkaline environment and sintered at 1200 °C for 2 h. It was reported that the obtained foamed ceramics had a porosity of ≈88.64% and a compressive strength of 0.47 MPa.^[^ ^19^ ^]^ Recently, many researchers have reported the synthesis of cordierite ceramics from different raw materials. These include sepiolite, diatomite, vermiculite silica fume waste, stevensite, and andalusite.^[^ ^20^, ^21^ ^]^ For thermal and electronic applications, composites containing strontium feldspar were prepared by solid‐state sintering of Sr‐feldspar/cordierite and (MgSrAl_4_Si_5_O_18_)/borosilicate glass powder mixtures. The results showed that Sr‐feldspar and cordierite crystallized in composites containing up to 20% borosilicate glass.^[^ ^22^ ^]^ Cordierite‐based composites include cordierite/feldspar, cordierite/alumina, cordierite/mullite, cordierite/borosilicate glasses, cordierite‐mullite‐corundum and cordierite‐ZrO_2_, etc.^[^ ^23^, ^24^ ^]^ Alkaline and alkaline feldspars are also in the important category of aluminum silicate materials that are largely applied for electronic applications.^[^ ^25^ ^]^

Although geopolymer materials are extremely stable on a chemical or microstructural scale after exposure to high temperatures, which can be considered a disadvantage of geopolymer materials. However, evaporation of water at high temperatures leads to an increase in capillary tensions within the geopolymer, resulting in significant shrinkage and cracking. Excessive shrinkage or cracking is highly unfavorable for engineering applications of geopolymers.^[^ ^26^, ^27^ ^]^ The effect of cations such as Na and K on shrinkage results in lower sintering temperatures. This is because Na is eutectic with alumina silicate at lower temperatures. In Na, the higher the cation content results in an increase in the shrinkage value. It has been shown that the shrinkage of Na‐based geopolymers is greater than that of K‐based geopolymers at the same temperatures.^[^ ^27^, ^28^ ^]^ Regardless of the type of alkali cation, the total shrinkage of fly ash‐based geopolymer was limited to 3%.^[^ ^29^ ^]^

The fact that exhaust treatment systems are not 100% sufficient and the high rate of vehicle costs have led to the development of studies in the automotive sector in this direction and the concentration of academic studies in this direction. The best example is cordierite, a porous oxide ceramic mineral. The advantages of this mineral such as high surface area, favorable active surface properties, and heat resistance have enabled studies in this field. This study is based on the production of geopolymer‐based cordierite, especially since the surface area of this mineral is desired to be high. By taking advantage of the properties of geopolymer structures such as having a porous structure, containing catalyst materials depending on the chemical recipe, being resistant to pressure and temperature, easy applicability, and being made from residual materials, it is envisaged to create geopolymer‐based cordierite.

## 2 Experimental Section

Open porous geopolymer‐based cordierite materials are produced due to the porous structure, temperature resistance, and easy and low‐cost applications of geopolymer structures, which are oxide ceramic materials that can act as natural catalysts for the emission treatment of diesel engines. For the composition of cordierite, waste boron clay, metakaolin, fly ash, and magnesium carbonate are used, while keeping the geopolymerization temperature constant, sodium silicate (NaSiO_3_), sodium hydroxide (NaOH), polypropylene and glass fiber, hydrogen peroxide (H_2_O_2_) are used to create an alkaline environment. These materials (filters) are tested in a 4‐stroke 4‐cylinder diesel engine's exhaust system at 50, 75, and 100 Nm engine torques and 1500, 1700, and 1900 rpm engine speeds.

### 2.1 Raw Materials

Metakaolin powder (MEFISTO L05) with an average particle size of 5 µm was used to prepare the suspensions. Type F fly ash with low calcium content was obtained from Kütahya Thermal Power Plant, boron waste clay raw materials were obtained from Kırka and Emet Plant Directorates, magnesium carbonate (MgCO_3_) and aluminium oxide (Al_2_O_3_) materials were obtained from Uşak Ceramic Factory. The chemical compositions of the raw materials used in the study are given in **Table** **1**. In addition to solid raw materials, sodium silicate Na_2_SiO_3_ (to create an alkaline environment), sodium hydroxide (NaOH) (also known as caustic soda), polypropylene and glass fiber, hydrogen peroxide (H_2_O_2_), calcium stearate (CaSt2), hexamine (as a surfactant), olive oil (to improve pore stability), Master Glenium 51 (polycarboxylic acid‐based superplasticizer) were used to improve processability, rheology and mechanical properties in suspension.

**Table 1:** Chemical composition of raw materials used in the study.

Chemical compounds	Raw materials [%]				
	Fly ash	Metakaoline	Boron waste clay	Magnesium carbonate	Aluminum oxide
SiO_2_	53.3	50.4	39.8	4.78	0.4
Al_2_O_3_	20.5	44.5	11.8	0.6	99.3
Fe_2_O_3_	9.3	0.53	4.7	0.78	0.016
MgO	4.18	0.04	6.72	74	0.048
CaO	2.16	0.15	7.62	2.46	0.015
K_2_O	1.84	0.12	4.4	0.07	0.017
TiO_2_	1.1	1.45	0.54	–	–
B_2_O_3_	–	–	9.75	–	–
Na_2_O	0.053	0.07	0.51	0.043	0.082
MnO	0.15	–	–	0.033	–
Cr_2_O_3_	0.178	0.02	–	0.02	–
NiO	0.25	0.06	–	0.04	–
CO_2_	6	2.56	–	14.2	–
Others	0.11	0.017	13.9	0.34	0.02

The raw materials used in the production of cordierite/nepheline minerals, which are geopolymer‐based oxide ceramic materials, are listed in **Table** **2**. Table 2 shows the percentages of raw materials used in the cordierite formulation and the variable values of hydrogen peroxide (H_2_O_2_) to provide porosity and alumina (Al_2_O_3_) to determine adsorption capacity.

**Table 2:** Raw material ratios and variables used in cordierite production.

Filter name	Raw materials and chemical components [%]					
	Fly ash	Metakaolin	Magnesium carbonate	Alumina	Boron waste clay	Hydrogen peroxide [mL]
	SiO_2_.Al_2_O_3_.Fe_2_O_3_	Al_2_O_3_.2SiO_2_	MgCO_3_	Al_2_O_3_	B_2_O_3 _Al_2_O_3 _SiO_2_	H_2_O_2_
1	44.14	33.25	19.04	1	5	2
2	44.14	33.25	19.04	2	5	2.5

### 2.2 Characterization Methods

The chemical composition of raw natural pyrophyllite‐rich clay (PY) was determined by X‐ray Fluorescence (Axios PANalytical spectrometer). The XRD data from the different geopolymer preparations were measured using a Bruker D8 Advance Twin diffractometer using KαCu irradiation at a wavelength of λ = 1.5418 Å. The diffraction patterns were determined over a 5–60° 2θ range, time of reckoning 0.3 s and step scanning of 0.05°. FTIR spectra were carried out in the range of 400–4000 cm^−1^ using the IRAffnity‐1S Shimadzu spectrophotometer. TGA and DTA analyses were performed using the Q500 TA instrument. The TGA and DTA phenomena of raw PY and PY‐GP4 samples were examined over a 25–1000 °C temperature range with a rate of 10 °C min^−1^. The specific surface area of raw PY and PY‐GP4 samples was determined using the BET method.^[^ ^25^ ^]^

### 2.3 Preparation of Geopolymer Formation

**Figure 2:**
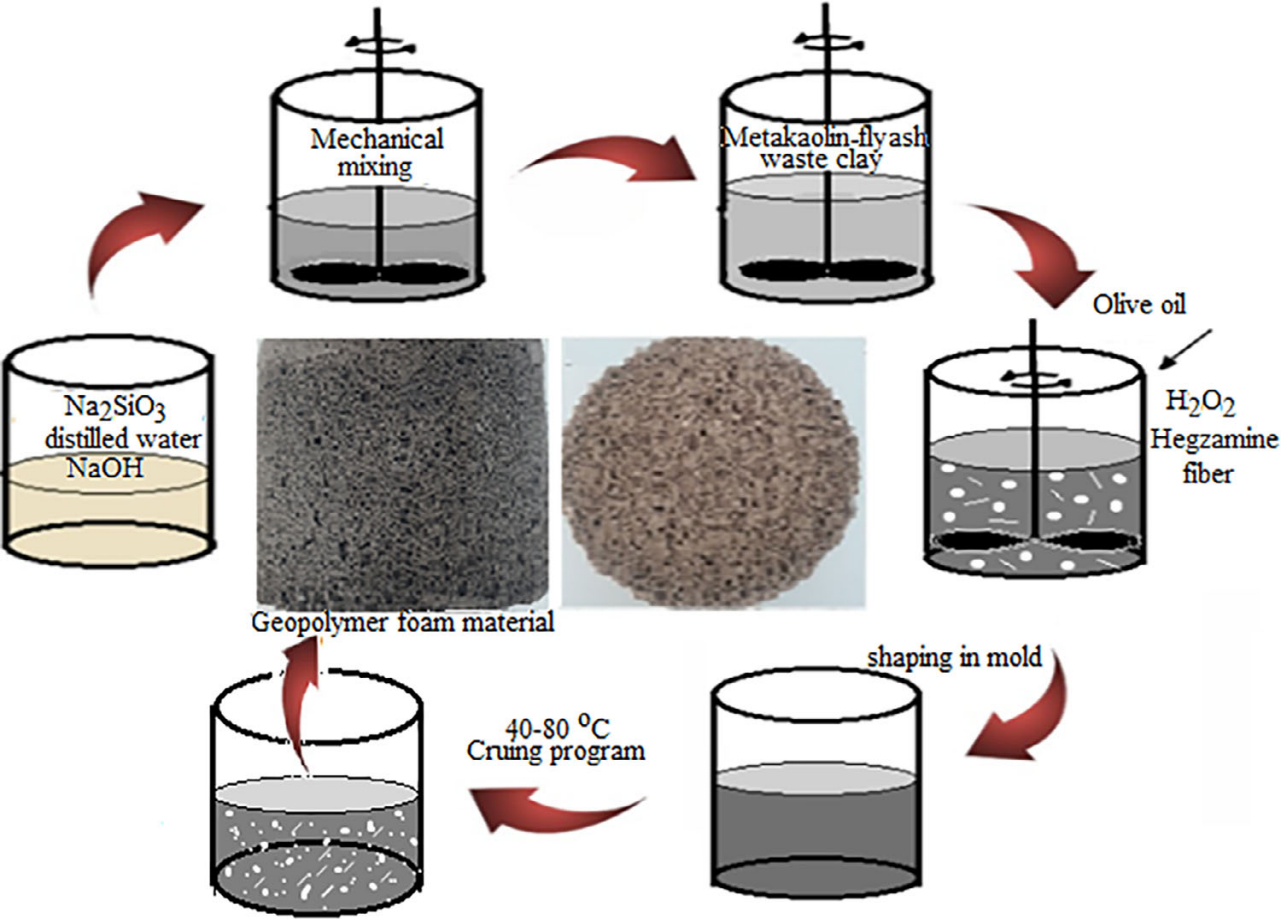
Experimental study process steps.

In the first step, the raw materials of the cordierite (Al_4_Mg_2_Si_5_O_18_) formulation determined according to Table 2 were weighed and homogeneously mixed. Alkaline mixtures were prepared at a predetermined molarity (6 M) by dissolving pieces of caustic soda in sodium silicate. To increase the strength, two different types of polypropylene and glass fibers were added to the alkaline mixture in predetermined amounts. The prepared alkaline mixture was poured onto the solid raw materials and mixed for 5 min. Foaming agent (H_2_O_2_), calcium stearate, and olive oil were then added to the geopolymer structure (mud consistency) to increase pore stability and mixed for a further 3 min. The mixture was poured into 150 × 150 mm cylindrical molds and cured in an oven at 70 °C for 24 h. The steps followed in the experimental studies are shown in **Figure** **2**.

### 2.4 Characterization Preparation Stage

**Figure 3:**
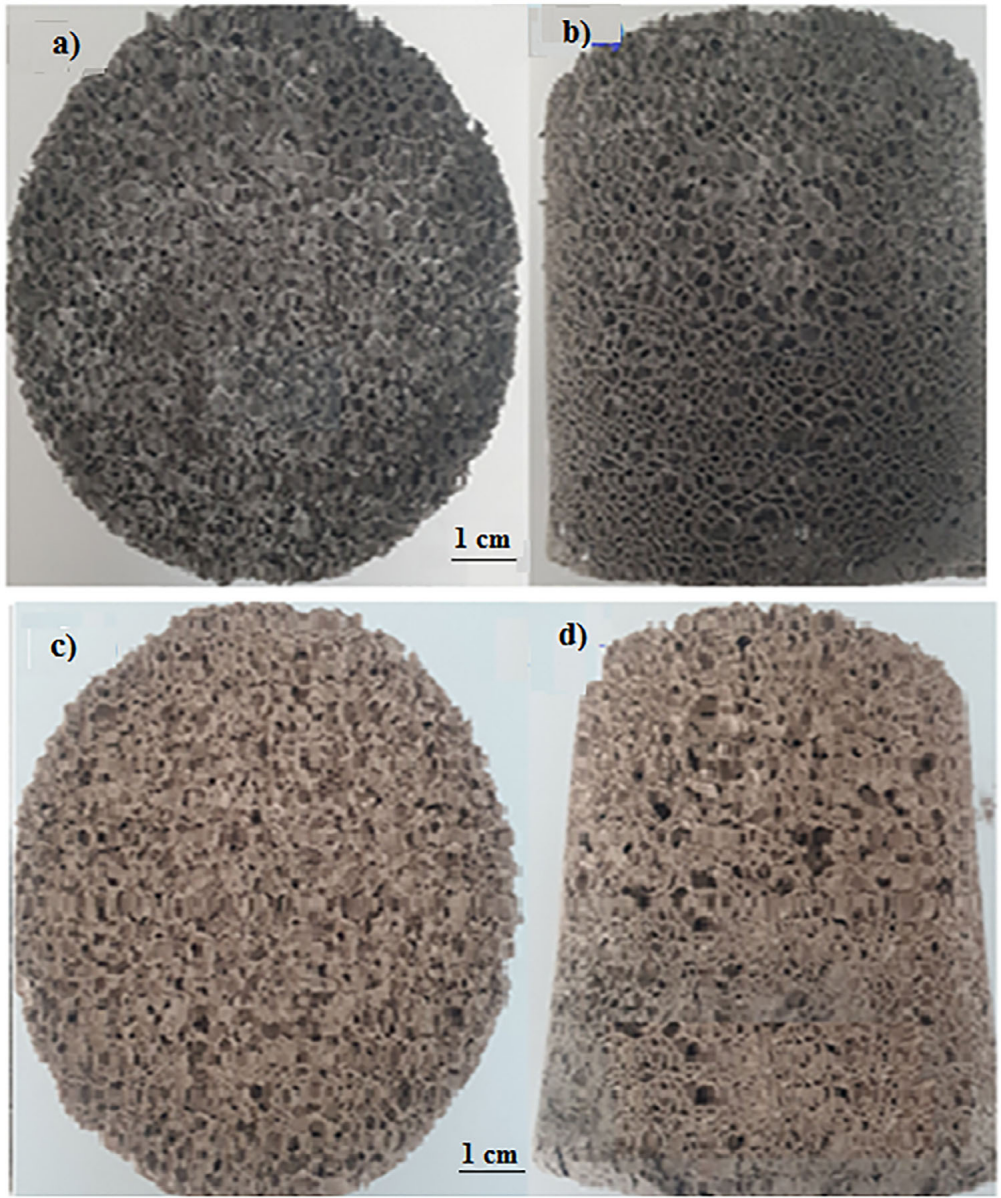
a) Top view of a geopolymer‐based porous material containing 2 mL of hydrogen peroxide before sintering, b) Front view of the same sample, c) Top view of the same sample sintered at 650 °C, d) Front view of the same sample sintered at 650 °C.

Samples were developed depending on the appropriate additions and process conditions to form a stable structure (foam) in geopolymer systems that show self‐curing by polycondensation at low temperatures. In the studies, 2 and 2.5 mL hydrogen peroxide were used as variables. The prepared filters were subjected to a curing program at room temperature for 28 days to gain strength. The strengthened filters were sintered in an oven heated up to 650 °C to increase their strength. Geopolymer material was realized in two different structures “before sintering” and “after sintering”. **Figure** **3** shows macro images of the first sample.

**Figure 4:**
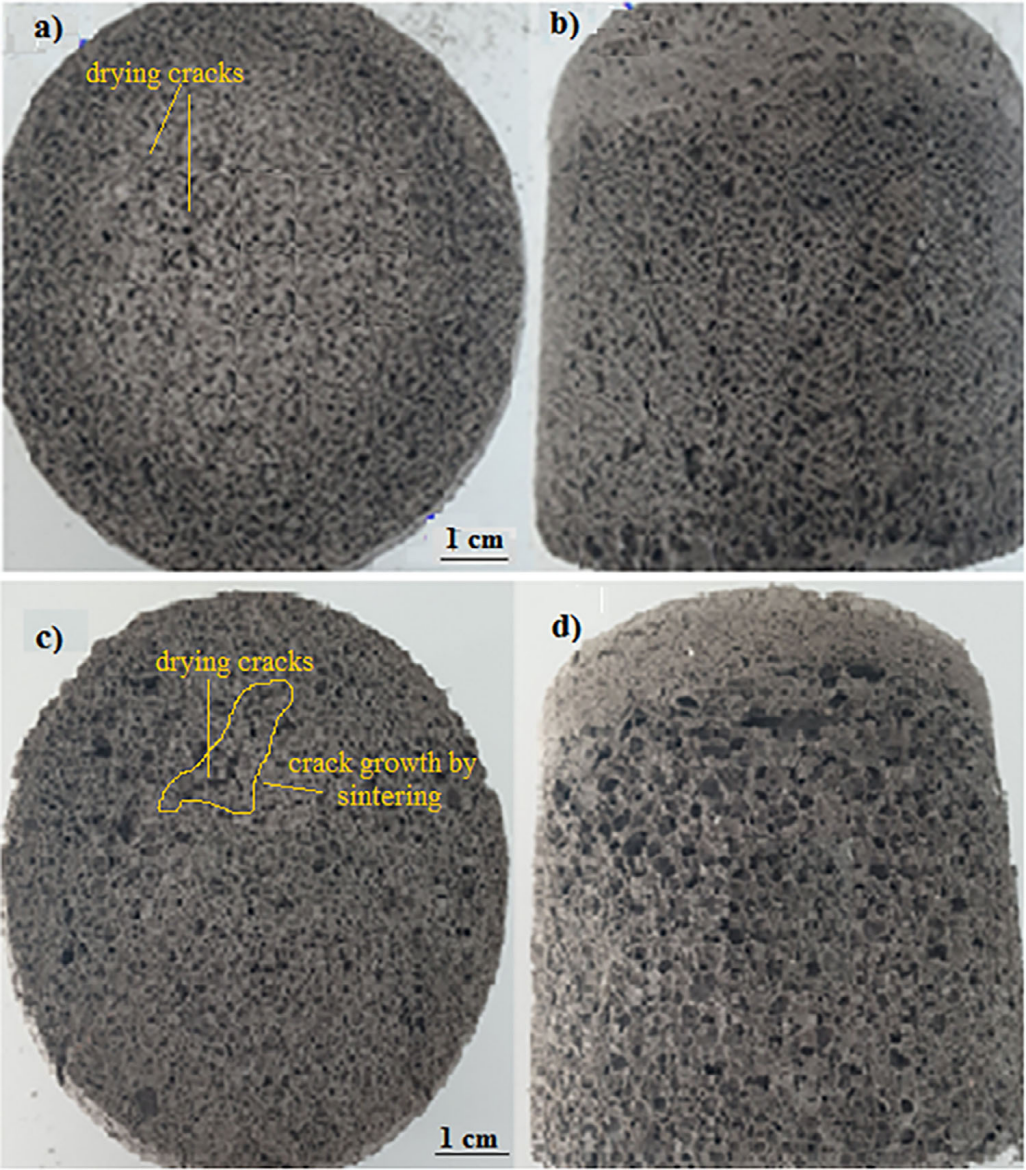
a) Top view of a geopolymer‐based porous material containing 2.5 mL of hydrogen peroxide before sintering, b) Front view of the same sample, c) Top view of the same sample sintered at 650 °C, d) Front view of the same sample sintered at 650 °C.

**Figure** **4** shows a macro view of the geopolymer‐based porous material in the sintered and unsintered form of the same sample with the addition of 2.5 mL of hydrogen peroxide (H_2_O_2_). Compared to the 2 mL hydrogen peroxide doped sample, an increase in the number of pores and a decrease in their density were observed. The main problem with geopolymer‐based porous materials is the formation of micro‐cracks due to drying. The size of these cracks increases with sintering.

## 3 Results and Discussion

### 3.1 Scanning Electron Microscopy (FE‐SEM)

**Figure 5:**
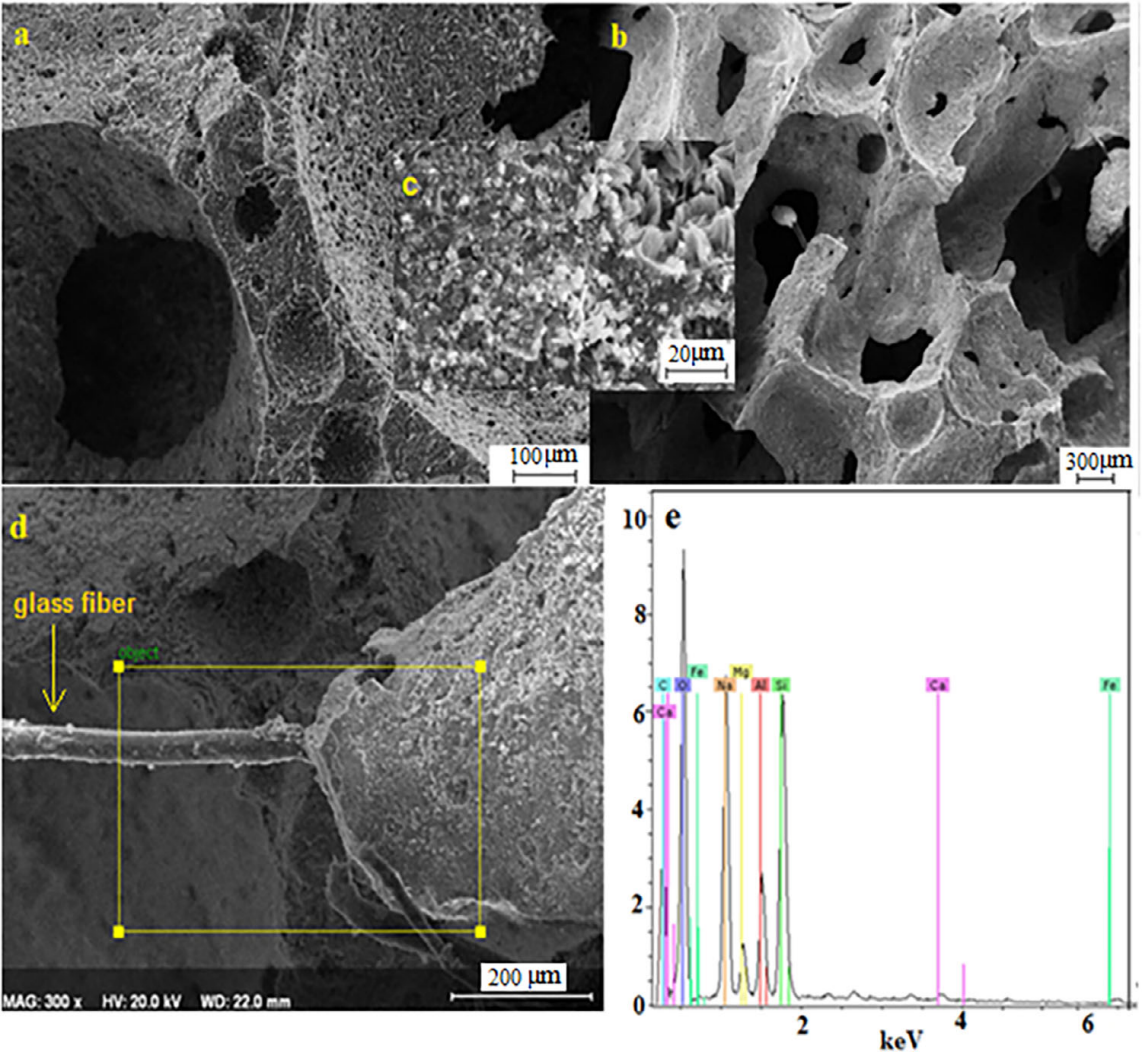
SEM and EDS images of the unsintered sample.

**Figure 6:**
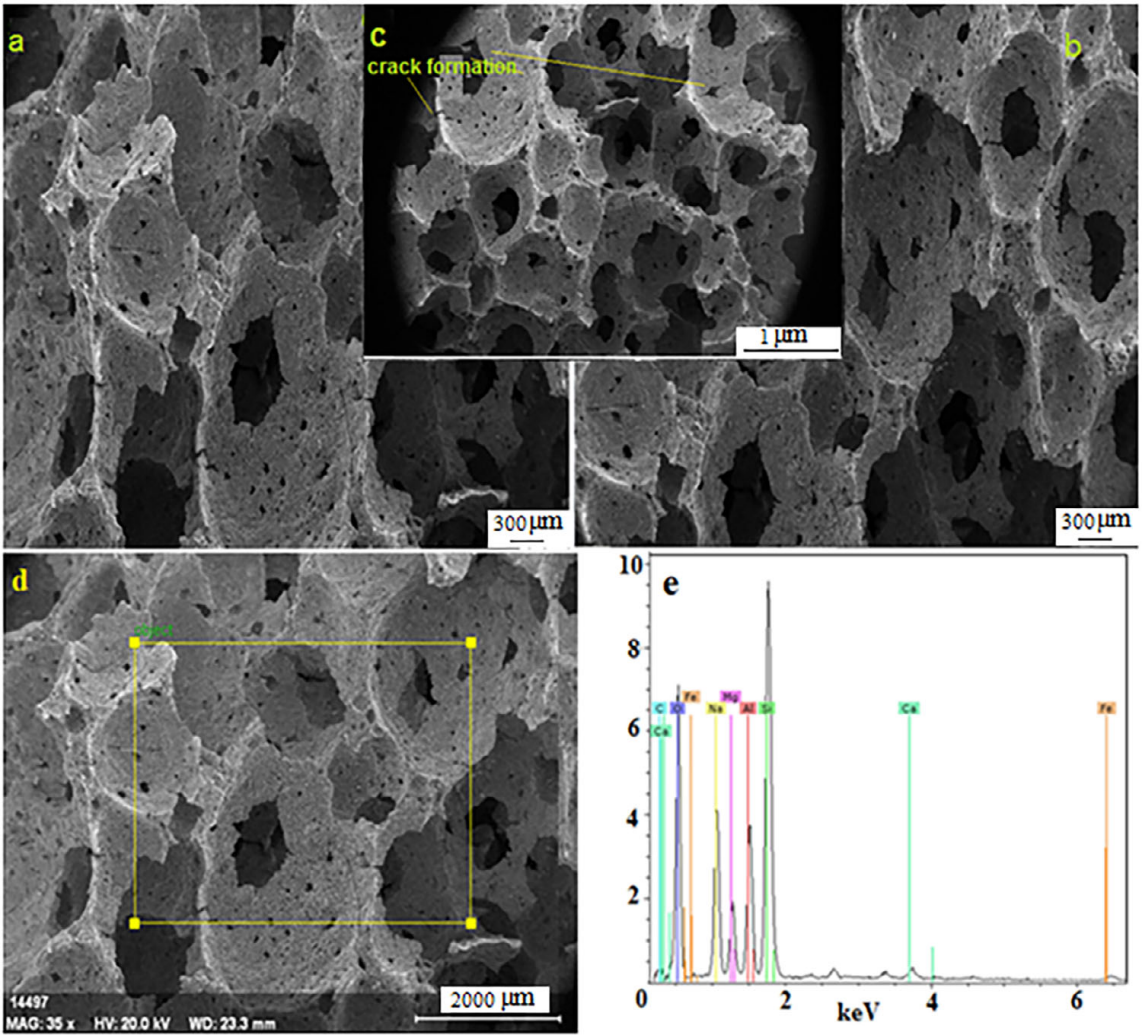
a–d) SEM image of the sintered sample e) EDS analysis images.

**Figure 7:**
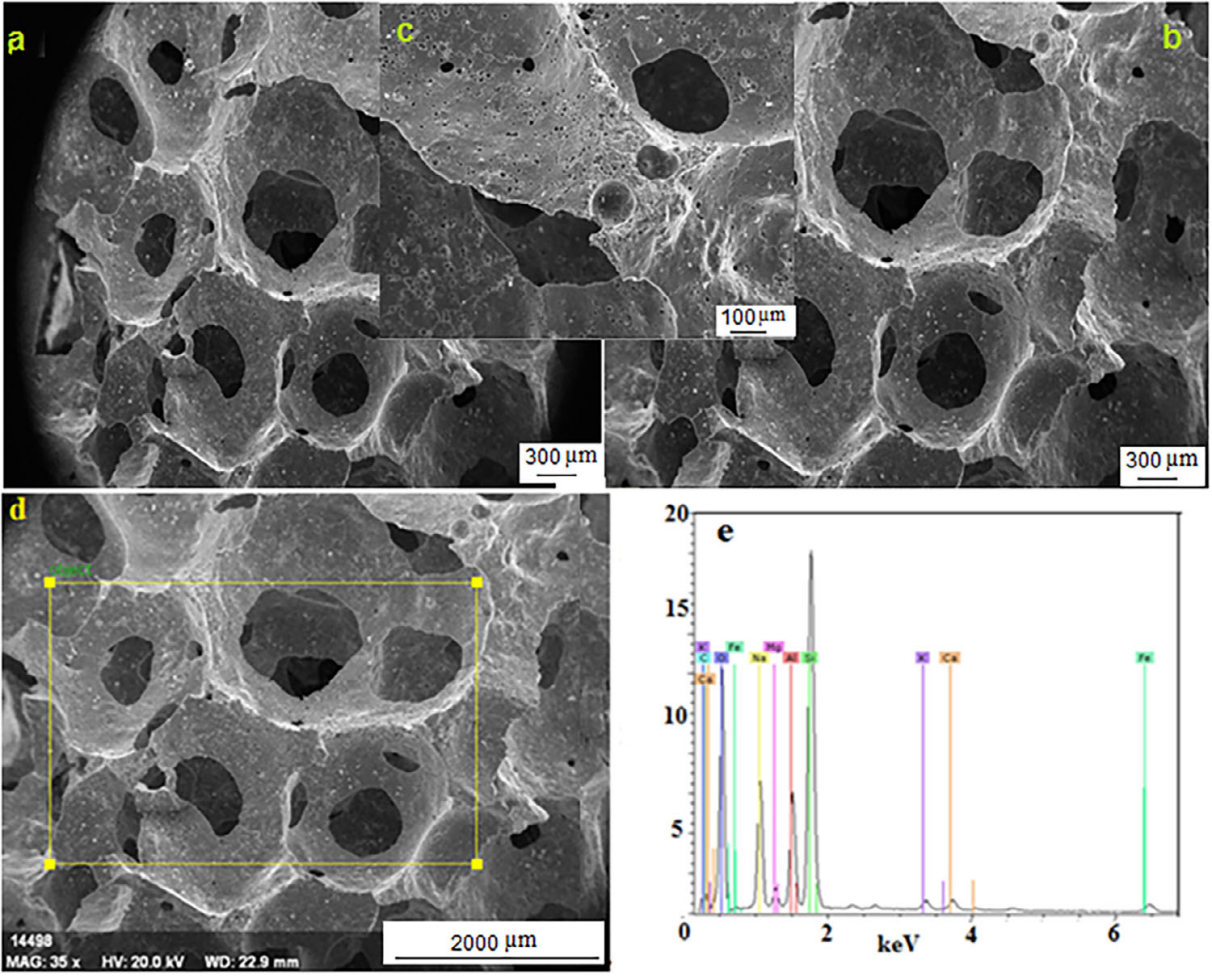
a–d) SEM image of the unsintered sample e) EDS analysis images of the same sample.

**Figure 8:**
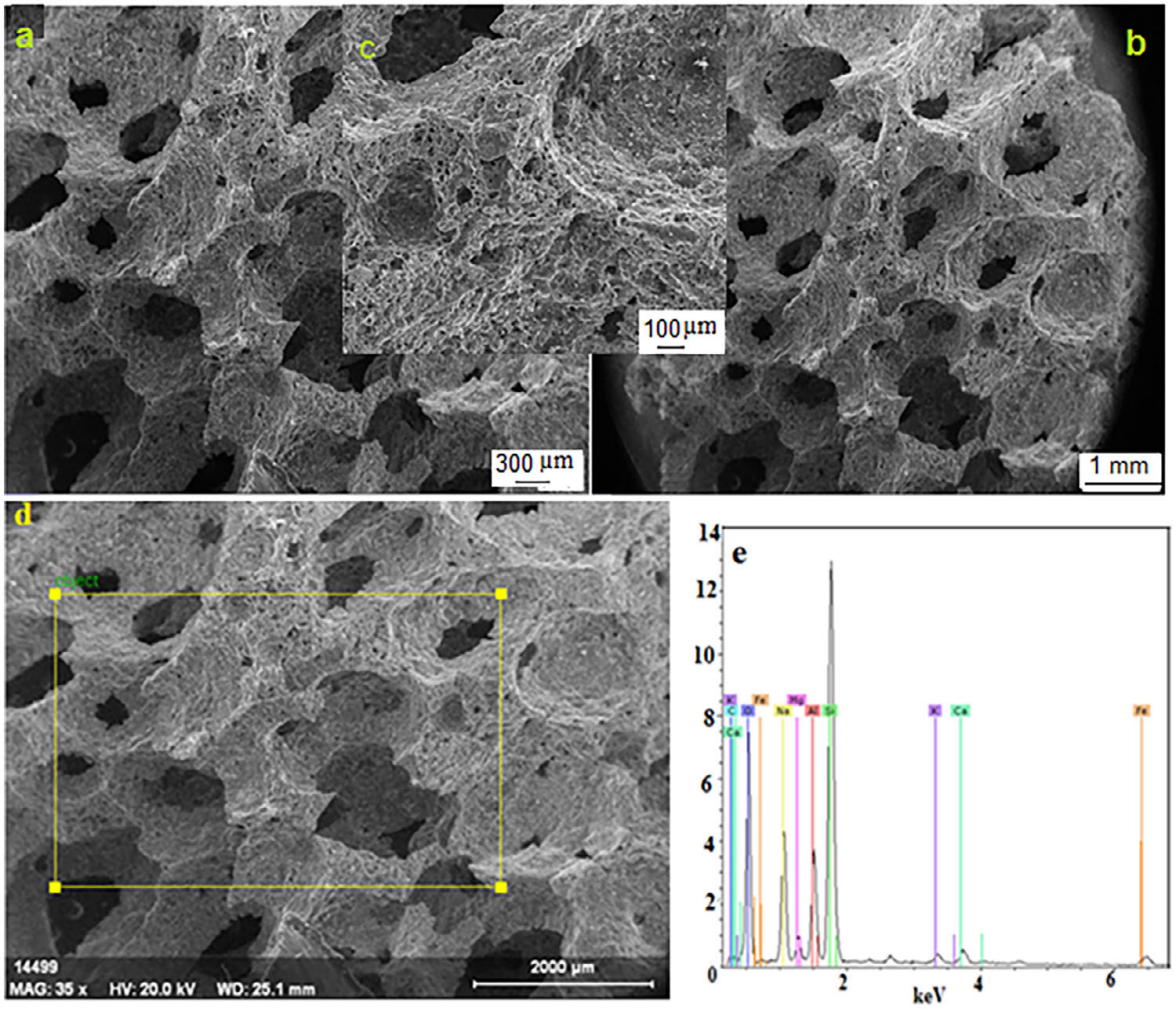
a–d) SEM image of the sintered sample e) EDS analysis images of the same sample.

The data obtained from scanning electron microscopy SEM and EDS analysis of geopolymer foam material are presented. **Figures** **5**, **6**, **7**, **8** SEM analyzes are given in two different images, sintered and unsintered. Figure 5a–e is data from the analysis of the unsintered sample. The wide range of pore size distribution was indicated in the BET analysis and these results were also visually revealed in the SEM analysis. While the increase in pore size distribution is an advantage for purification and filter isolation purposes, it is a disadvantage in terms of strength. In Figure 5a, we can see pores in two different size ranges. One ranges in size from microns to 0.3 mm, while the other ranges in size from A° to microns. The interconnection of the pores and their networked relationship provides an advantage in terms of use for different purposes, but a disadvantage in terms of strength. Figure 5 shows the images of glass fibers used as reinforcement material in d samples and their connections with the matrix material. The glass fibers were used to increase the strength at the same rate in all samples. Most of the pore cells were not spherical but appeared as interconnected network systems of cell windows. There is also the formation of independent closed pores. The best example is shown in Figure 5c. There are many parameters affecting the pore size in geopolymer structures. Some of them are NaOH, water, calcium stearate, and chemical prescription which makes it alkaline. In the images obtained by SEM, Figure 5d,e shows the presence of O, Mg, Si, Al, Na, and minor elements Fe, Ca, and Cl in the light of semi‐quantitative information obtained from the surfaces of the samples examined with the help of EDS spectrum.

Figure 6a to e shows the SEM and EDS analyses of the same sample sintered at 650 °C. BET pore analysis revealed that the pore size and quantity decreased with sintering. In the SEM data taken from these samples, it is seen that the pore sizes decrease in Figure 6a–d samples. It was revealed that some cracks and pores in the structure became larger. The BET analysis also showed that while the total amount of pores decreased with sintering, the pore size distribution increased. Larger pore distributions show weaker brittle properties in terms of strength at the connection points of approximately 300 µm. This facilitates the formation of microcracks as in Figure 6c. Figure 6e shows the presence of major elements O, Mg, Si, Al, Na, and minor elements Fe, Ca, and Cl in the light of semi‐quantitative information obtained from the surfaces of the samples examined with the help of EDS spectra.

Figures 7 and 8 show the SEM and EDS analyses of the same sample as unsintered and sintered, respectively. Figure 7a–e is data from the analysis of the unsintered sample. Figure 7a–c shows an irregular, unsymmetrical, and very wide distribution of pore sizes. The pore size distributions are quite wide and irregular. While the increase in pore size distribution provides an advantage for filter, absorption, and isolation, it is a disadvantage for the strength of the porous material. Figure 7c,d shows the SEM image of the same sample at different magnifications. Here, irregular pores are seen more clearly and grain boundaries are more distinct. Figure 8 shows the sintered SEM and EDS images of the same sample. Although there is some decrease in pore sizes with sintering, the majority of the pore volumes can be seen. In e, EDS analysis of the same sample was taken. The analysis shows the elements forming cordierite/nepheline and other phases. The presence of O, Mg, Si, Al, Na, Mg, Si, Al, Na, and minor elements Fe, Ca, and Cl are observed. Drying cracks are observed at the grain and pore boundaries and sometimes inside the grain. Most pore cells are not spherical but appear as interconnected cell windows (network systems). There is also the formation of independent closed pores.

In unsintered samples, the decrease in the size of stable pores formed during geopolymerization causes problems during drying. These cracks can occur during drying cracks or sintering. These cracks are the most common problem in geopolymer foam materials. The evaporation exit velocity from the surface of the geopolymer and the exit velocity from the material (volume) should be equal or the exit velocity from the surface should be slow. The way to overcome these problems is to increase the pore size and/or increase the strength to withstand the evaporation stress.

### 3.2 Multipoint BET Analysis

**Figure 9:**
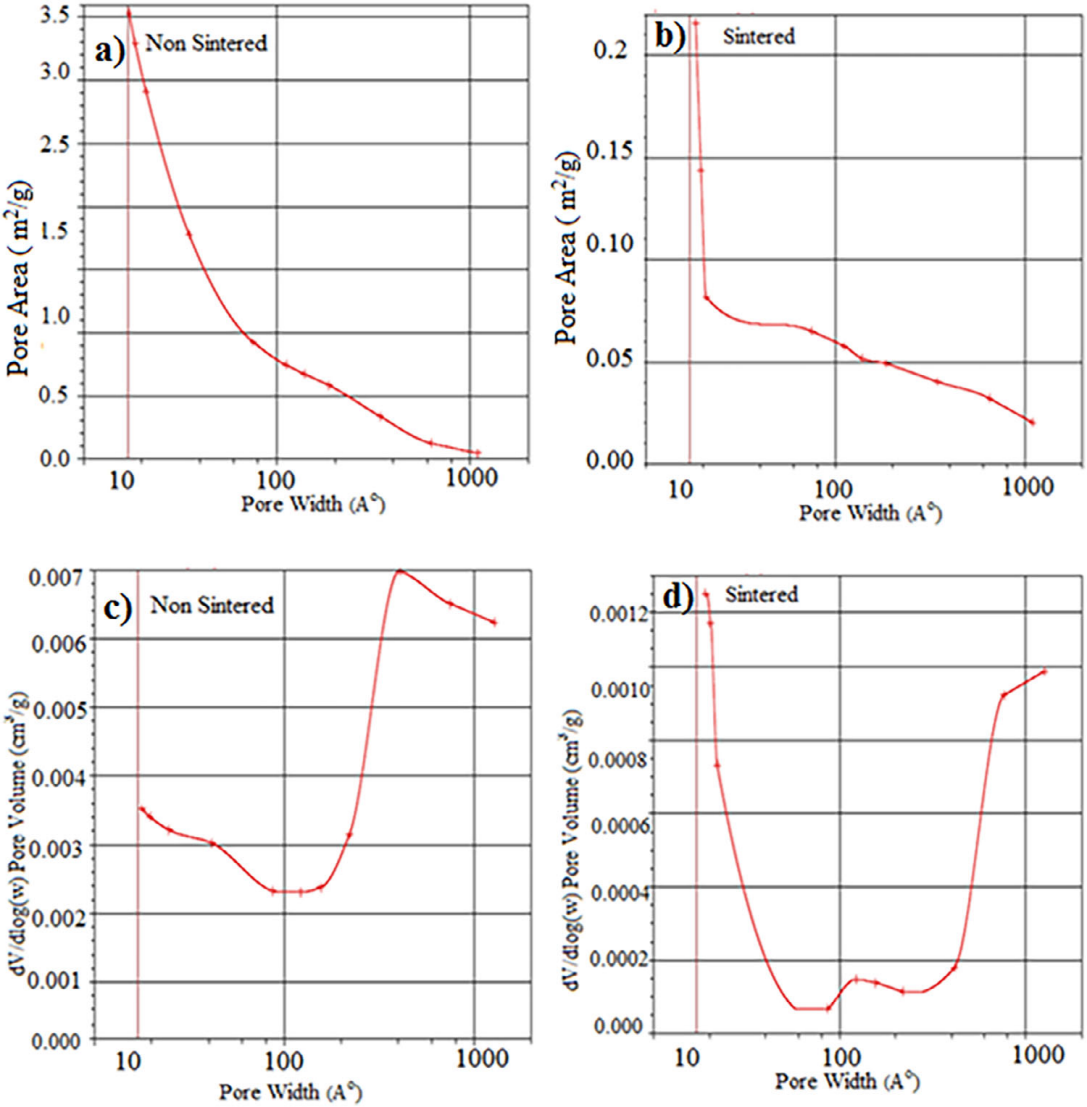
a,c) Multipoint BET analyses of unsintered, b,d) Sintered samples.

BET Analysis: In **Figure** **9**a–d, pore surface area (m^2^ g^−1^)‐ Pore size Angtrom (A° = 10^−8^ cm) and Pore volume (cm^3^ g^−1^)‐ Pore size (width A°) analyses are given for unsintered and sintered oxide structured porous materials of the same sample whose pore distributions were examined by BET analysis. While the pore size distribution in Figure 9 shows a very wide distribution of 10–1000 A°, the unsintered sample in Figure 9a has a surface area of 0.5 × −3.5 (m^2^ g^−1^). With sintering, the pore size distribution remains the same in the sample in Figure 9b, while the surface area of the pore decreases with this pore size distribution. Figure 9c,d shows the differentiated volume‐pore size distribution for the unsintered/sintered samples. The larger pore size occupies more volume in the unsintered sample. In the sintered sample in Figure 9d, there is a decrease in the volumes with larger pore sizes. In both sintered and unsintered samples, most of the pore size volume in both sintered and unsintered samples consists of pores distributed in the range of 200–800 A°. It shows that there is no regular pore distribution. In Figure 9a, the specific surface area of the pores was determined as 3.5 (m^2^ g^−1^). As the range in the pore size distribution changes, the specific surface area also changes. The larger the pore size, the smaller the specific surface area. This shows that the distribution of pore sizes is small and medium. p/p° = 0.252113371 single point surface area: 4.3916 m^2^ g^−1^. It is seen in all samples that the pore size decreases with the sintering of the same samples and the pore surface area decreases accordingly.

**Figure 10:**
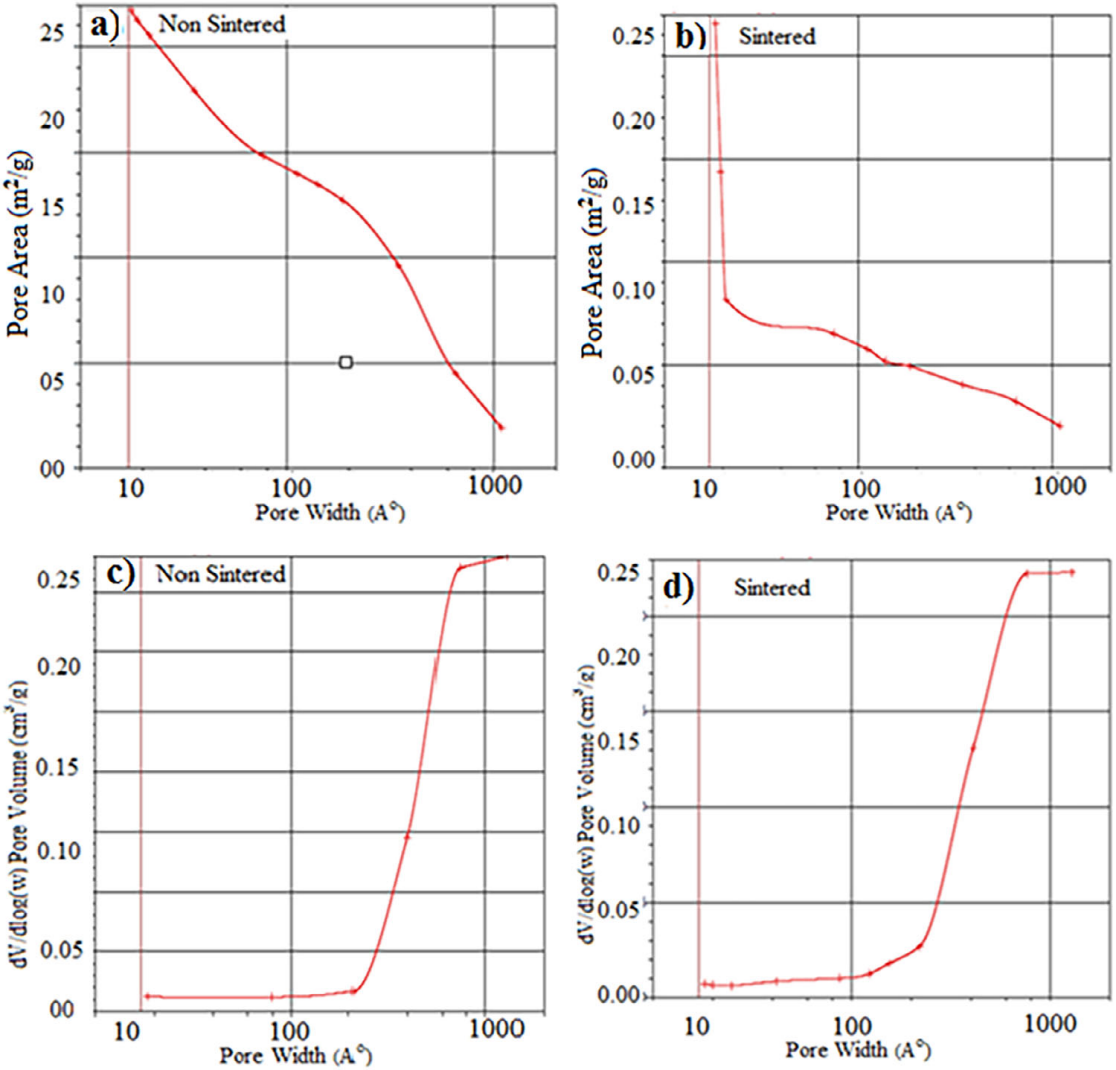
a,c) Multipoint BET analyses of unsintered, b,d) Sintered specimens.

**Figure** **10**a–d shows the pore surface area (m^2^ g^−1^) pore size (A°) and pore volume (cm^3^ g^−1^) pore size (width A°) analyses of unsintered and sintered oxide structured porous materials of the same sample formed by increasing the amount of Hydrogen peroxide in the cordierite recipe. a) Adsorption cumulative pore volume between 15.000 and 3 000 000 A° width: 0,0 09257 cm^3^ g^−1^, pore size adsorption average pore diameter 82654 A°, Desorption average pore diameter 82303 A° Adsorption average pore width 104808 A° were measured. Single point surface area at p/p° = 0.252097281 in the analysis from the unsintered sample in Figure 10a,c: 17.4963 m^2^ g^−1^ BET Surface Area: 19,2645 m^2^ g^−1^ Cumulative surface area of adsorption pores between 17000 and 3 000 000 A° in width: 217 272 m^2^ g^−1^.

The curves in Figure 10 show a bimodal pore distribution in general, but the pore sizes vary between sintered and unsintered. It is seen that a decrease of up to 30% in pore surface area is realized with sintering. It created a change in the amount of micro and meso pores. No noticeable changes were observed in the amount of macro‐sized pore size. With the sintering of the same samples, it is seen in all samples that the pore size decreases and the pore surface area decreases accordingly.

### 3.3 XRD Analysis

**Figure 11:**
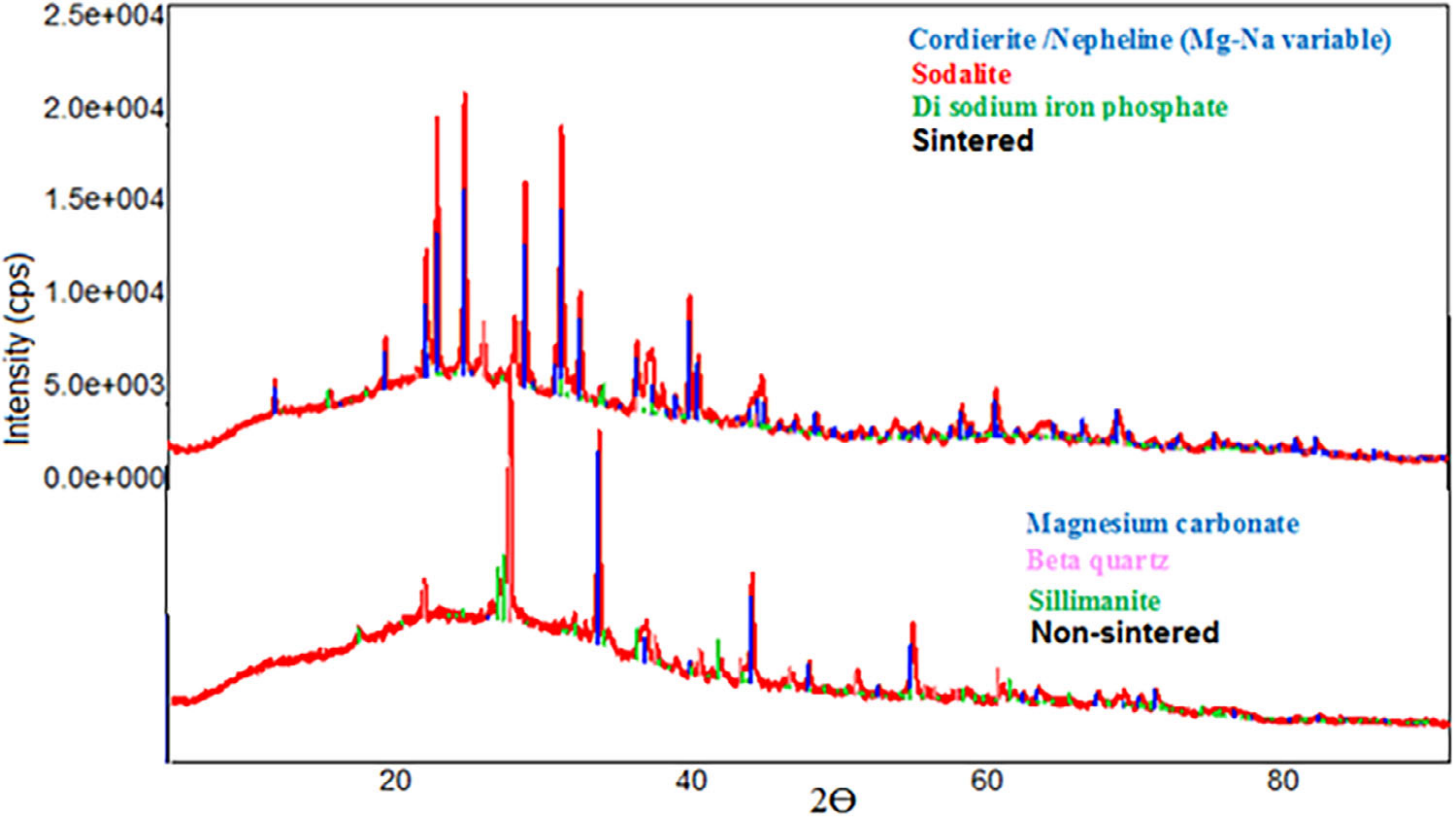
XRD peaks of sintered and unsintered samples.

**Figure** **11** shows the XRD analysis peaks of sintered and unsintered samples. Looking at the unsintered samples, it is seen that most of the magnesium carbonate reacts and turns into Cordierite. In addition, it is seen that Na from the alkaline environment (Base and Glass water) is transformed into Nepheline. The peaks of nepheline and cordierite (Mg and Na variables) intersect in most places. Sodalite in the sintered sample appears to be a different component of sodium alumina silicate. Another Na component has also been compounded with iron and phosphate entering as an impurity to form Di sodium iron phosphate. Sillimanite (Aluminum silicate) is almost completely transformed into Cordierite/Neferin and Sodalite. In the unsintered sample, it is seen that it is transformed into nepheline rather than cordierite formation together with the residual quartz.

### 3.4 TGA and SDT Analysis

TGA analysis was performed using the SDT Q600, an instrument capable of measuring both thermal and mass changes simultaneously. A small amount of sample (≈20 mg) was placed in a platinum crucible and heated at a rate of 10 °C from room temperature to 800 °C min^−1^. The sample was exposed to a nitrogen gas flow of 100 mL min^−1^ to prevent oxidation. Temperature and mass readings were calibrated using pure metals and alumina with known melting points.

**Figure 12:**
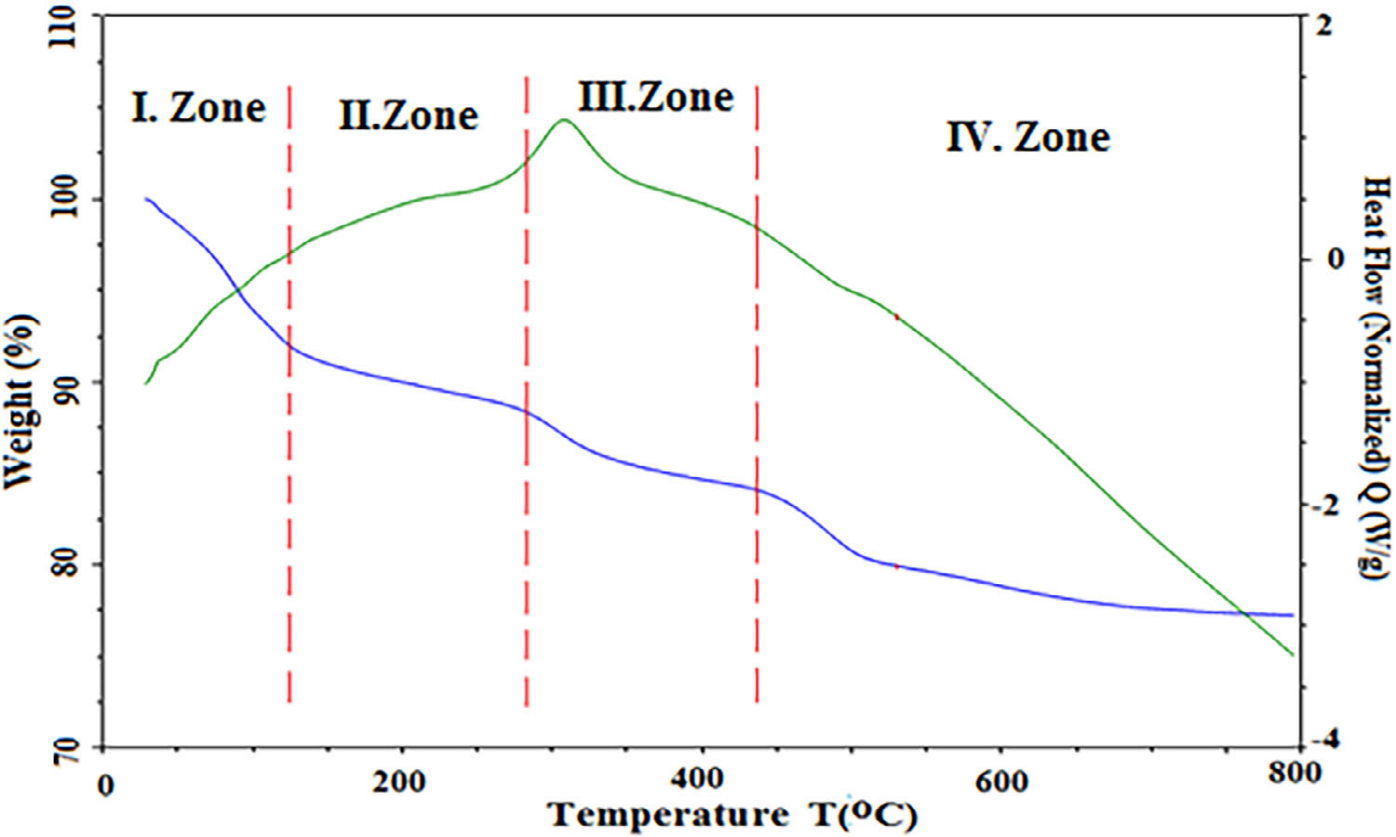
TGA and SDT analysis.

In **Figure** **12**, temperature zones I, II, III, and IV are between 20 and 120 °C, 120 and 300 °C, 300 and 500 °C and 500 and 800 °C, respectively. The weight loss geopolymer material showed the presence of aluminosilicate gel. The weight loss of the minerals in the aluminosilicate gel in the I. region‐ which occurs with sintering temperatures‐ is related to evaporation. Pore water, followed by dehydroxylation (zone‐II) involves the loss of water bound to the hydroxyl group at 100 to 300 °C. It is the mass loss that leads to a decrease in density and causes the geopolymer network to collapse. It accounts for approximately 12% mass loss. During the reaction in Zone III, the combination of dehydroxylation with the formation of an aluminosilicate gel leads to shrinkage of the microstructure and transformation of the mineral composition into new mineral phases.^[^ ^30^, ^31^ ^]^ This occurs exothermically at 300–350 °C. In temperature zone IV, a continued viscous sintering process significantly reduces porosity densifies the thermal shrinkage, and further homogenizes the matrix.

**Figure 13:**
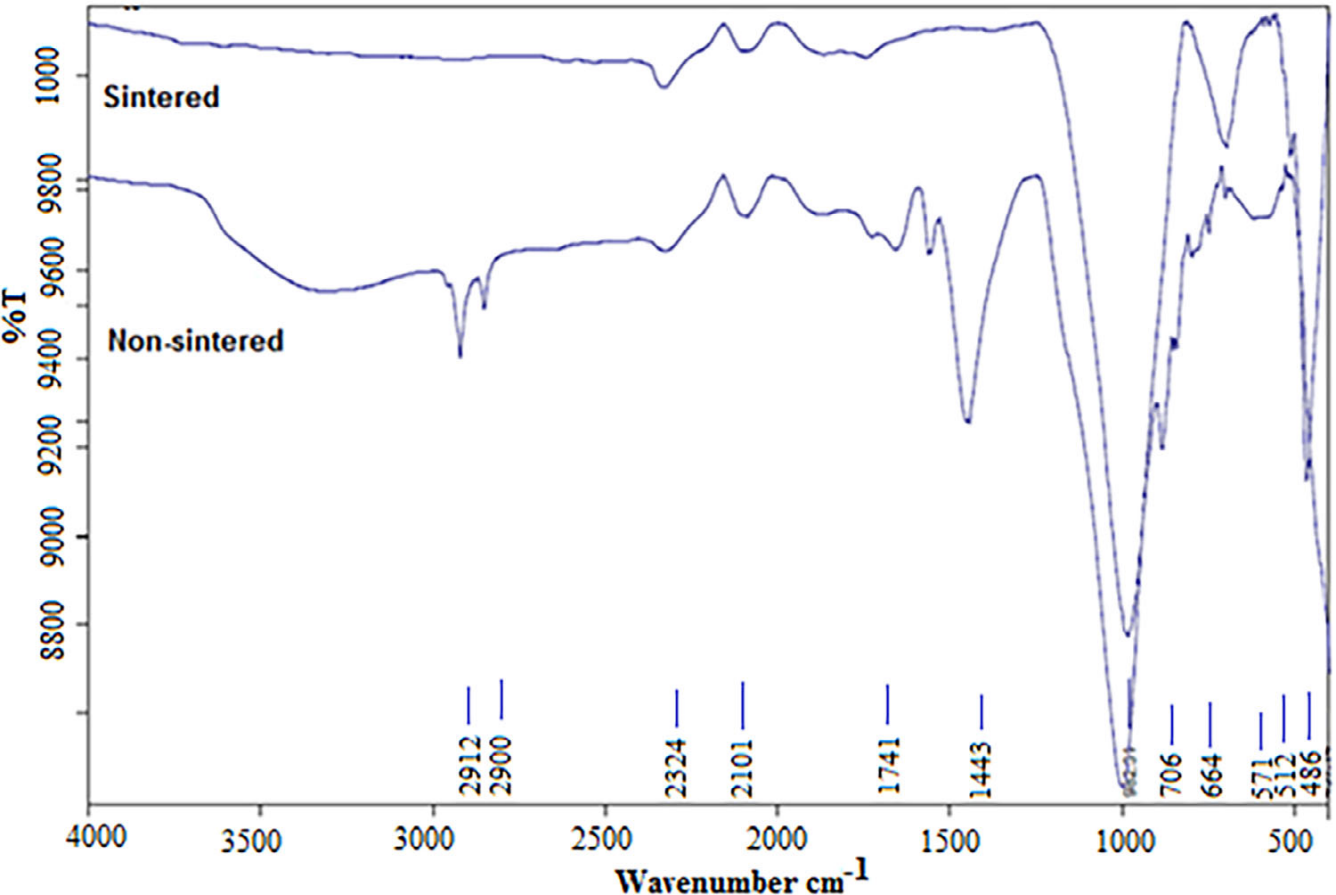
FTIR analysis of sintered and unsintered geopolymer samples.

**Figure** **13** FTIR Spectrometer: sintered and unsintered. The main peaks seen between 1000 and 600 cm^−1^ are known to be associated with asymmetric vibrations of K─O─Si bonds (K = Si or Al).^[^ ^32^, ^33^ ^]^ The peak at 970–800 cm^−1^ is associated with the bending vibrations of the Al─O─Si bond and the peak at ≈420, 980 cm^−1^ is associated with the Si─O─Si bending vibration.^[^ ^34^ ^]^ In geopolymer compositions with organic surfactant added, the small fluctuations seen in the 2200–1800 cm^−1^ wave number section indicate symmetric and asymmetric vibrations of CH_2_ due to the organic surfactant content.^[^ ^33^ ^]^

The intensities of the observed peaks vary depending on the amount of functional groups in the synthesized geopolymer. At the same time, shifts to different wave numbers in the peaks are due to the inclusion of a different atom in the gel matrix.^[^ ^35^, ^36^, ^37^ ^]^ The broad and sharp vibrational bands at 3000 and 1650 cm^−1^ indicate the stretching and bending of H─O─H bonds and the asymmetric stretching of O─C─O at 1454 cm^−1^, respectively. The vibration peaks seen here are related to the water molecules contained in the geopolymer sample or remaining in the pores. It is an indication that more water molecules may be present in the pores with the enlargement of the pores. It is possible to observe that the increase in the H─O─H peak actually expands the pore structure, a lower‐density material is obtained and as a result, the strength values decrease. It shows that H─O─H bonds disappear with sintering. The peak intensities of the functional group in geopolymers may differ from their densely produced geopolymer counterparts. The chemical bonds visible in the FTIR bands correspond to the molecules found in the XRD results and indicate that the geopolymer is properly activated. Shifts in wavelength and an increase in the area under the peak with increasing peak intensity are indicative of increased cross‐linking. Larger aluminosilicate polymer molecules with a higher degree of polymerization lead to better mechanical properties.^[^ ^38^, ^39^ ^]^

### 3.5 Exhaust Application of Cordierite Formed by Geopolymer Method

To investigate the adsorption effects of Al_2_O_3_ on exhaust emissions, the developed filters were installed separately in the exhaust treatment system of the diesel engine. With the filters installed, the diesel engine was operated under 9 different operating conditions with torques of 50, 75, and 100 Nm and engine speeds of 1500, 1700, and 1900 rpm. The emissions of CO, HC, NOx, and particulate matter, which are harmful exhaust products resulting from the operation of the diesel engine, were measured. In order to determine the oxidation and filtration properties of the filters used for CO, HC, NOx, and particulate matter, emission data was collected at two points “before” and “after” the filters. The results of the exhaust emissions at the measurement points of “before filter” and “after filter” are shown in **Table** **3**. “Before filter” results show the engine out data without any emission treatment system, while “After filter” results show the treated emission data.

**Table 3:** Results of the exhaust emissions at the measurement points of “before filter” and “after filter”.

Engine torque	Engine speed	Filters	Test point	CO	HC	NO_x_	Particulate matter
[Nm]	[rpm]			[% Vol]	[ppm]	[ppm]	[mg/m^3^]
100	1500	1. Filter	Before filter	0.57	25	691	0.21
			After filter	0.48	18	651	0.15
		2. Filter	Before filter	0.54	23	852	0.23
			After filter	0.22	12	810	0.03
	1700	1. Filter	Before filter	0.33	29	694	0.16
			After filter	0.24	19	654	0.07
		2. Filter	Before filter	0.31	20	649	0.15
			After filter	0.15	14	619	0.00
	1900	1. Filter	Before filter	0.15	29	694	0.12
			After filter	0.09	15	643	0.07
		2. Filter	Before filter	0.15	22	677	0.10
			After filter	0.10	7	643	0.00
75	1500	1. Filter	Before filter	0.11	22	662	0.10
			After filter	0.07	16	569	0.02
		2. Filter	Before filter	0.11	31	655	0.09
			After filter	0.07	11	591	0.01
	1700	1. Filter	Before filter	0.07	28	715	0.02
			After filter	0.05	15	539	0.00
		2. Filter	Before filter	0.08	22	615	0.02
			After filter	0.04	15	510	0.01
	1900	1. Filter	Before filter	0.07	26	515	0.01
			After filter	0.04	16	477	0.00
		2. Filter	Before filter	0.07	23	538	0.02
			After filter	0.04	14	496	0.00
50	1500	1. Filter	Before filter	0.06	25	437	0.01
			After filter	0.04	19	404	0.00
		2. Filter	Before filter	0.07	21	560	0.04
			After filter	0.04	8	426	0.00
	1700	1. Filter	Before filter	0.09	29	399	0.01
			After filter	0.04	22	366	0.00
		2. Filter	Before filter	0.09	23	423	0.05
			After filter	0.03	9	377	0.03
	1900	1. Filter	Before filter	0.10	28	410	0.01
			After filter	0.06	14	354	0.00
		2. Filter	Before filter	0.11	24	374	0.03
			After filter	0.05	8	277	0.00

**Figure 14:**
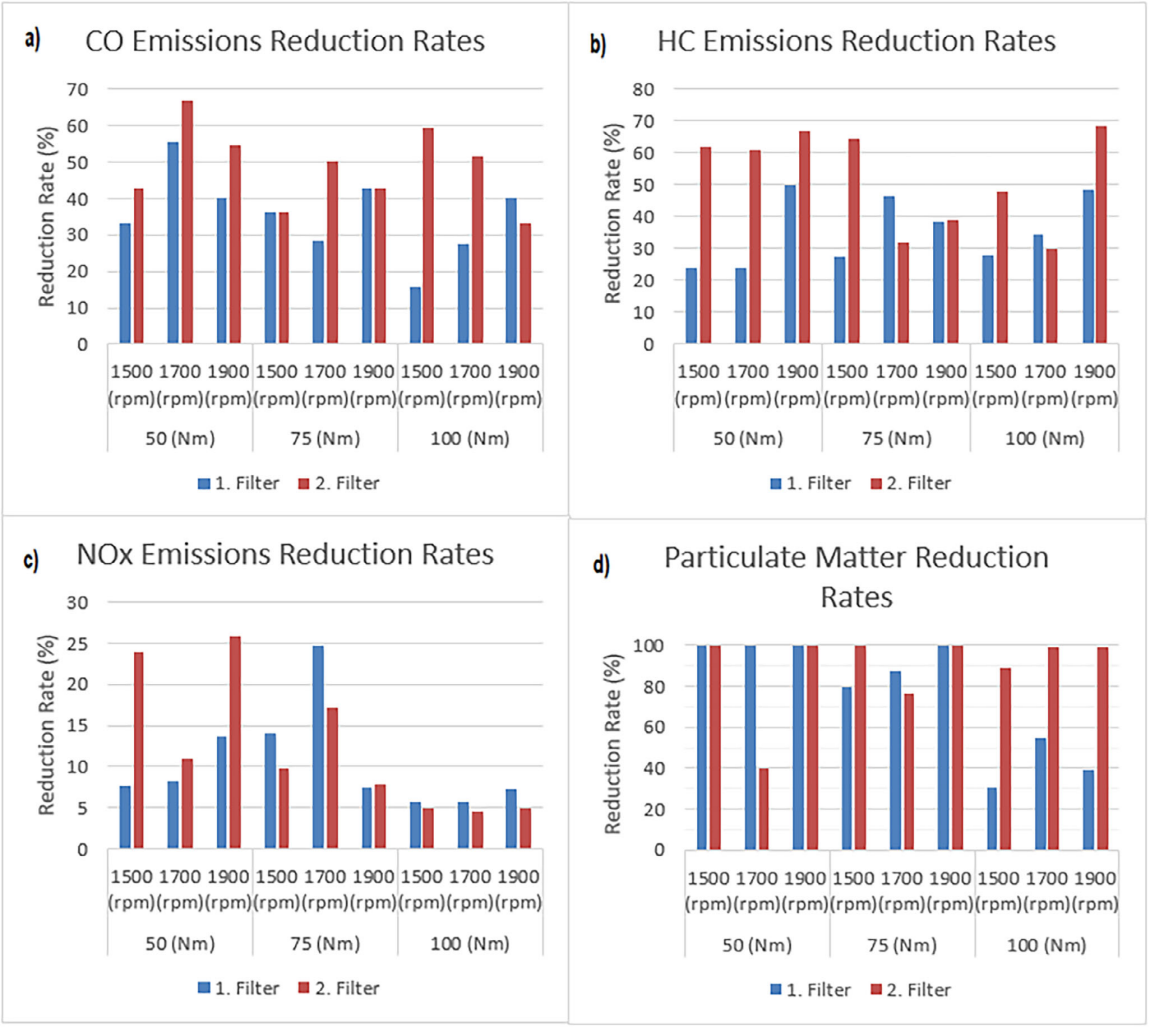
Emission reduction rates by using the filters.

By comparing the values measured “before” the filter with the values measured “after” the filter; the rate of reduction in exhaust emissions due to the use of filters was analyzed in terms of CO, HC, NOx, and particulate matter. The emission reduction rates by using the filters are shown in **Figure** **14**.

The rates of reduction in CO emissions as a result of the filters oxidizing the CO emissions by acting as a catalyst are shown in Figure 14a. While the 1st filter reduces CO emissions by 16–55%, the 2nd filter reduces CO emissions by 33–66%. This is due to the higher amount of Al_2_O_3_ in the 2nd filter compared to the 1st filter, which enables the 2nd filter to oxidize CO emissions better and has the ability to reduce CO emissions.

The percentages of HC emission reduction from the filters as a result of using the filters in the exhaust treatment system are shown in Figure 14b. With the use of the 1st filter, the highest HC emission reduction rate is found at 48%. This rate was obtained with the diesel engine running at 1900 rpm with a torque of 100 Nm. With the use of the 1st filter, the lowest reduction rate of HC emissions is determined as 24%. This rate is observed at engine speeds of 1500 rpm and 1700 rpm with a diesel engine torque of 50 Nm. With the use of 2nd filter, the highest reduction in HC emissions is calculated to be 68% at 1900 rpm and 100 Nm torque, and the lowest reduction is found to be 30% at 1700 rpm and 100 Nm torque. This shows that the 2nd filter is more capable of reducing HC emissions compared to 1st filter.

The percentage reduction in NOx emissions with the use of filters in the exhaust treatment system is shown in Figure 14c. The NOx emission reduction rates of both filters are between 5 and 25%. The NOx emission occurs due to high exhaust temperature which is a normal phenomenon for internal combustion engines. The high temperature of exhaust gases oxidizes nitrogen to NOx. The developed material in this research is used in the exhaust final line after the engine is out, so, its material's effect on the reduction of NOx emission is limited. Because the NOx emission is related to the high exhaust gas temperature.

The reduction percentages of particulate matter obtained as a result of the utilization of filters are shown in Figure 14d. The 2nd filter retains the particulates by filtering >95% at all engine torques and speeds. On the other hand, as the engine torque increases, the 1st filter's particulate retention decreases. This shows that the 2nd filter is more effective in terms of its ability to retain particulate emissions and performs the filtering task at a better rate than the 1st filter.

Cordierite/nepheline oxide minerals produced by the geopolymer process consist of tetrahedral and octahedral structures which make the surface more active. As a result of the easy displacement of metals in these structures, a more active surface is formed. This feature is important for the adsorption of exhaust gases.

## 4 Conclusion

This study is based on the production of open‐cell cordierite by geopolymer method in the utilization of waste raw materials, and the effect of hydrogen peroxide was experimentally investigated. The product is developed for the emission treatment of diesel engines which play an important role in the environmental effect. As the main product of the study, open‐cell (oxidation catalyst) geopolymer‐based minerals consisting of porous cordierite/nepheline phases were obtained. Waste fly ash, waste boron clay, metakaolin, and glass fiber were used in the production of these minerals. With the use of these materials, the possibility of utilizing industrial wastes and by‐products has been created and properties that can be used for different purposes have been gained. The developed materials (filters) are tested in a 4‐stroke 4‐cylinder diesel engine's exhaust system at 50, 75, and 100 Nm engine torques and 1500, 1700, and 1900 rpm engine speeds to show the reliability of the products. The main conclusions of the study can be given as follows:

1. Cordierite with open porosity was obtained with an average density of 0.28 g cm^−3^ and porosity over 70%.
2. As the amount of hydrogen peroxide increases, the amount of porosity increases, but the strength decreases significantly.
3. Porous materials are often used in many industrial applications such as filtration, absorption, catalysis, lightweight structural materials, and insulation materials. Geopolymer foam (porous) materials have been initially prescribed and realized based on the structure of cordierite (2MgO 2Al_2_O_3_ 5SiO_2_). As a result, this material is recommended to be investigated for exhaust filtration, purification, absorbent, and insulation purposes.
4. Cordierite/Nepheline minerals produced via the geopolymer method exhibit superior thermal stability, low thermal expansion, and high resistance to thermal shocks. These properties make them ideal for applications in high‐temperature environments, such as diesel exhaust systems or thermal insulation.
5. Cordierite/Nepheline oxide minerals produced by geopolymer method are composed of tetrahedron and octahedron structures, which make the surface more active. This feature provides an important feature for the adsorption of exhaust gases.
6. The use of open‐cell geopolymer materials (filters) reduced CO emissions by up to 66% and HC emissions by up to 68%.
7. The use of the developed exhaust after‐treatment filters in the diesel engine reduced NOx emissions by up to 25%.
8. The open‐cell geopolymer materials were found to be effective in treating over 95% of particulate matter.

The 2nd filter containing 2% Al_2_O_3_ was found to be more effective than the 1st filter in terms of its ability to retain exhaust gases and solid particles. It is concluded that the developed products are useful tools for the emission treatments of diesel engines considering the oxidizing and filtering effects of the materials.

## Conflict of Interest

The authors declare no conflict of interest.

## Data Availability

The data that support the findings of this study are available from the corresponding author upon reasonable request.
